# Sentence contexts and cloze probabilities for Brazilian Portuguese children and adolescents

**DOI:** 10.1371/journal.pone.0236388

**Published:** 2020-07-30

**Authors:** Natalia Freitas Rossi, Catarina Fernandes, Célia Sofia Moreira, Célia Maria Giacheti, Bianca Bortolai Sichieri, Ana Patrícia Pinheiro, Adriana Sampaio

**Affiliations:** 1 Faculty of Philosophy and Sciences, São Paulo State University (UNESP), Speech, Language and Hearing Science Department, Campus of Marília, São Paulo, Brazil; 2 Psychological Neuroscience Lab, CIPsi, School of Psychology, University of Minho, Braga, Portugal; 3 Faculty of Sciences and Center of Mathematics (FCUP & CMUP), University of Porto, Porto, Portugal; 4 Faculty of Psychology, University of Lisbon, Lisbon, Portugal; Nagoya University, JAPAN

## Abstract

**Purpose:**

In this study we investigated a set of 100 sentence contexts and their cloze probabilities to develop a database of linguistic stimuli for Brazilian Portuguese children and adolescents. The study also examined age-related changes on cloze probabilities, and specified the predictor effects of age and cloze probabilities on idiosyncratic responses and errors (semantic, syntactic, and other errors). Finally, the study also aimed to shed light on cultural effects on word generation by comparing Brazilian and Portuguese sentence databases.

**Method:**

361 typically developing monolingual Brazilian speakers, with ages ranging from 7 to 18 years, participated in the study. The cloze task was composed by 100 sentence contexts, grounded on the European Portuguese database. Responses were classified as valid (correct) or invalid (semantic, syntactic, and other-type errors). Statistical analyses were based on mixed-effects logistic models.

**Results:**

Sixty-three sentences met criteria for high cloze probabilities, 30 for medium cloze, and 7 for low cloze. Age was a significant predictor of idiosyncratic responses, semantic and syntactic errors: older participants were less likely to produce idiosyncratic responses, as well as semantic and syntactic errors. Cloze probability values were concordant in the Brazilian and Portuguese databases for 31 out of 49 (83.7%) high-cloze sentences and for 7 low-cloze sentences.

**Conclusion:**

In this study we have provided a database with cloze probability values for a set of 100 sentence-final word contexts for Brazilian Portuguese children and adolescents. Results showed that both age and sentence contextual level predicted sentence final word completion. Older participants were more likely to choose more consistently the same final word, with the contextual level of a given sentence also contributing to the final word selection. Age should be controlled for in future studies probing semantic processing with this set of sentences.

## Introduction

The language comprehension system changes across development and depends on a complex interaction between neurobiological and sociocultural factors [1, 2]. It is not surprising that sentence comprehension is one of the most sophisticated cognitive abilities of the human brain [3], relying on a well-orchestrated interplay of several cortical brain regions [4]. Specifically, the brain must recognize a word and its lexical form (spoken or written), assign meaning to that word (semantic processing), retrieve specific grammatical rules (syntactic processing), and relate the meaning to its broader context (pragmatic processing). Language comprehension is, therefore, one of the major challenges that children must master to become proficient language users, remaining a fundamental cognitive ability throughout life [3].

In particular, semantic processing refers to the ability to access acquired knowledge and to make sense out of otherwise arbitrary linguistic symbols [5], which relies on critical interactions with the semantic memory system. Semantic memory represents the internally organized knowledge about word meanings and their relationships [6, 7], as well as the conceptual knowledge of the world [6]. Therefore, it is essential for language comprehension [8], and for the functional use of language [9].

Sentence comprehension recruits a distributed network of brain regions, including the left inferior frontal gyrus, the right superior temporal gyrus, the left middle temporal gyrus, and the left posterior temporal region [10]. The selection and retrieval of lexical representations is supported by the inferior frontal cortex, whereas the anterior temporal cortex and angular gyrus support the integration of lexical input into the larger units of sentences or discourse [11]. Although this network is observed at 7 years, substantial neurodevelopmental changes occur underpinning semantic processing until adulthood [12].

Classical models of semantic processing [13–15] highlight the importance of semantic context in sentence comprehension, as the meaning of a sentence is more than the sum of the meanings of its individual words [16]. Contextual effects on language comprehension are observed very early in development [17], since context is required to solve ambiguities in sentence structure [2]. Sentence completion tasks have been used to probe oral and written language comprehension [18–20] in typically developing individuals and clinical populations [21–23]. Specifically, in the cloze task, participants are asked to complete a sentence with the first word that comes to their mind [24]. This task allows computing a cloze probability value for the final word that completes a given sentence context, which represents the proportion of individuals who provide the same final word to the sentence given the preceding words [18, 20, 25, 26].

In this context, Bloom and Fischler’s (1980) [27] sentence completion norms have been widely used in different languages, such as British English [28], French [29], Latino-American Spanish [30] and European Portuguese [31], and with different age groups [30, 31]. Additionally, cloze probabilities have supported the selection of stimuli in controlled experimental tasks with event-related potentials [18]. Despite the widespread utility of the Bloom and Fischler’s (1980) norms [27], some studies evince differences in cloze probabilities among different languages [28] and age groups [32, 33], which highlight the need to address cross-cultural comparisons and different age intervals. Accordingly, cultural and linguistic specificities such as level of education or vocabulary and grammatical rules are among the most cited limitations [27]. A robust body of evidence demonstrated that cultural contexts are critical factors that modulate language production and comprehension [34] and influence which semantic information is learned, used [35] and organized [36]. Neurophysiological studies also supports an interaction between cultural identity and semantic processing during sentence comprehension [3].

Of note, most sentence completion norms are restricted to adults [13, 18, 28, 29, 32, 33]. To the best of our knowledge, only two studies relying on cloze tasks have tested children and adolescents, with Mexican Spanish (ages of 9 and 12) [30], and European Portuguese (ages of 6 and 11) samples [31]. Even though Rodríguez-Camacho et al. (2011) [30] did not explore age effects, the authors highlighted the need to consider developmental processes in word recognition processes and language comprehension as databases relying on norms for younger and older adults are inappropriate to assess school-age children, who use less complex grammatical structures.

In the current study, sentence contexts were grounded on the European Portuguese database [31] for two main reasons: first, Portuguese is the official language in both countries (Brazil and Portugal), ensuring lexical and grammatical similarities but with different cultural contexts; second, the European database was developed for the target population of children and adolescents. Notwithstanding, even though Brazilian Portuguese shares a large part of the European Portuguese language heritage, some cultural specificities determine lexical differences between European and Brazilian Portuguese [37], including phonetic-phonological, morphological and syntactical levels [38]. Specifically, the pronominal system (“você” *vs*. “tu”), use of gerund *v*s. infinitive verb (“está chovendo” *vs*. “está a chover”), and morphological derivation (“desenhista” *vs* “desenhador”) are some of the main differences between Brazilian and European Portuguese [37]. Lexical differences include the use of different words for the same referent (e.g., “autocarro” *vs*. “ônibus” [bus]), the use of words with different meanings (e.g., “cueca” [underpants] *vs*. “calcinha” [panty]) and the presence of words derived from indigenous and African languages in Brazil [39]. Social and cultural factors may, therefore, influence the frequency of word use. For example, fish (“peixe”) and soup (“sopa”) are everyday food items used in Portugal whereas bean (“feijão”) and rice (“arroz”) are more commonly used in Brazil [39]. These social and cultural effects are observed also when comparing the psychometric properties of vocabulary assessment tools in Brazil and Portugal, with differences regarding normative data being observed for Brazilian Portuguese and European Portuguese [40]. These examples highlight the need to adapt stimulus material to each cultural context [40, 41] and are consistent with previous studies documenting modulatory effects of cultural and linguistic differences on cloze probability values [27, 28]. Although the main purpose of this study was not to provide a robust analysis of cultural-specific patterns (Brazil and Portugal) on cloze probabilities values, we have investigated the concordance between the Brazilian and the European Portuguese database in terms of cloze probabilities value.

Regarding developmental changes, Pinheiro et al. (2010) [31] described an age-effect for the cloze probability values, syntactic and semantic responses errors and idiosyncratic responses. There was a reduction in the numbers of idiosyncratic responses and semantic errors; and, nonetheless, and an increased consistency in the type of appropriate final words chosen to complete a specific sentence context. Authors argued that these findings support the idea that a very early mechanism is learned for extracting regularities in the environment and constructing common representations about things, indicating that consistency in word selection tends to increase during development.

In addition to cultural specificities of the cloze probabilities values, age was also considered an important variable to account for the current study for several reasons: 1) there is evidence that semantic networks mature during childhood and adolescence, which is reflected in age-related differences in language performance [42]; 2) previous behavioral studies have documented more consistent patterns of sentence context completion as a function of increased age [31–33], with a decreased number of idiosyncratic (i.e., words chosen by only one subject) and invalid responses (i.e., words that do not properly match their sentence contexts) [31]. Our prediction was that older participants would be less likely to generate idiosyncratic responses and errors (syntactic and/or semantic or other errors) due to advantages related to brain maturation, literacy, education and metalinguistic competences. In addition, we expected to identify more idiosyncratic responses and errors in younger participants for low-cloze probability sentences. This prediction is consistent with developmental changes in cognitive control (i.e., executive attention and inhibitory mechanisms) and sentence-level semantic processing that operate together with sentence constraint in determining word choice. This hypothesis implies that higher cloze probabilities would be associated with high-constraint sentences and enhanced executive control (leading to inhibition of other lexical possibilities) in older participants. The consolidation of lexical-syntactic and semantic processes in older individuals is expected to lead to advantages in the selection of a more accurate final word for a given sentence [31–33], reflected in an increased consistency in word selection [31], as well as in a decreased number of idiosyncratic (i.e., words chosen by only one subject) and invalid responses (i.e., words that do not properly match their sentence contexts) [31].

In sum, the primary goal of this study was to investigate a set of 100 sentence contexts and their cloze probabilities to develop a database of linguistic stimuli for Brazilian Portuguese children and adolescents, which was grounded on a European Portuguese database [31]. The current study also examined age-related changes on the most frequently selected sentence ending, as well as the predictor effects of age and cloze probabilities on the generation of idiosyncratic responses and errors (semantic, syntactic, and other errors). Finally, the study also aimed to provide a comparison of sentence constraint levels in the Brazilian and European Portuguese databases.

## Materials and methods

### Participants

Participants were 361 monolingual Brazilian Portuguese students with ages ranging from 7 to 18 years (*M* = 9.94 *SD* = 2.33; 171 females; 190 males): 290 were children aged from 7 to 11 years (*M* = 9.01, *SD* = 1.09) and 71 were adolescents aged between 12 and 18 years (*M* = 13.73, *SD* = 2.19). The following inclusion criteria were used: (a) age raging from 6 to 18 years old; (b) parent authorization with signed consent form; (b) no previous experience of grade repetition; (c) no current history of poor performance in reading, writing, and mathematics; (d) no reported behaviour problems or spoken language difficulties by the teacher and confirmed by a speech-language pathologist; (e) ability to complete the task in the written modality; and (f) correct answers in at least three of five training blocks. The study was approved by Ethical Committee of Faculty of Philosophy and Sciences, São Paulo State University (UNESP), Campus of Marília, São Paulo, Brazil (process number 0526/2012). All guardians signed written consent forms.

First, all teachers received information about the main goals of the current study. Second, the informed consent was sent to 490 parents of students from primary to secondary school levels. Parents of 431 students signed the informed consent. For those, the teacher was requested to fill out a questionnaire regarding their students’ language (oral and writing) abilities, academic performance, behaviour and communication problems. From 431 consented students, 410 fit the inclusion criteria. Four speech-language pathologists individually screened these students in a 15-minutes session that aimed to exclude major speech and language problems. From these 410 students, 5 (6 years old) were excluded and referred for a more specific evaluation due to speech sounds distortions (3 of 5) and speech fluency problems (2 of 5). Finally, the cloze task was administered to 405 students: 23 did not complete the task, 14 failed in the training block (<three corrected answers), and 7 were not able to complete the task in the written modality (all students with 6 years old). The final sample was composed of 361 participants. Participants’ socioeconomic level ranged from B1 to D, according to the Brazilian Association of Research Companies [43].

### Procedure

Data were collected in a quiet room at the school where participants were recruited from March to June 2014. The cloze task was composed by 100 sentence contexts: 73 were selected from the Pinheiro et al. (2010) database with European Portuguese participants [31], and the remaining 27 sentences were developed following the procedure described in this prior study. All sentences had the same sentence length (four words per sentence) and syntactic structure (subject and direct verb in the present tense: SVO). When a target word (subject or verb) was not available or it was unusual in the local cultural vocabulary, it was replaced by another word with semantic equivalence. Some adaptations were necessary for 19 of the 73 sentence contexts from Pinheiro et al. (2010) database [31]. The 100 sentence contexts are described in Appendix Table 1 in S1 Appendix. The 100 sentences were presented in written form (recording sheet), with text printed in black color on white paper (Times New Roman font, size 12). The recording sheet was composed of two columns (the stimulus column and column response) with 100 lines (one line for each sentence).

Participants were told that this was a task in which they would read sentences that were incomplete; they were instructed to complete them with the first word that came to their mind and that provided an appropriate ending for that sentence context. Specifically, the following instruction was provided: "*You will read sentences that are incomplete*, *that is they are missing a word at the end of the sentence*. *You will have to read carefully each one and complete the sentence with the first word that comes to your mind*. *This word must fit the rest of the sentence*, *that is*, *you will try to form a sentence that makes sense*. *Do you understand*? *Got it*? *Before we begin*, *a short practice session will take place*”. The training block included five sentences that were not part of the experimental task, ensuring that participants were familiarized with the instructions and with the task. During training, interventions by the experimenter were permitted to allow the participants to fully understand the nature of the task. These interventions included alerting to the semantic (i.e., a final word choice leading to a semantic violation as in “The seamstress uses the *whistle*”) and syntactic nature of potential errors (i.e., gender or number agreement with the object) or even to distraction, as well as providing positive feedback for appropriate words and negative feedback for inappropriate word selections. Participants were instructed to respond carefully since they were not allowed to use rubber.

### Response coding

Responses were coded as valid or invalid, following Pinheiro et al. (2010) [31]. A valid final word was computed if it completed the sentence context appropriately for both levels, semantic and grammatical. Additionally, for each valid response we computed (1) the frequency of the valid final word for a given sentence context to determine the proportion of participants who generated the same final-word sentence (cloze probability); (2) idiosyncratic responses (valid words generated by a single participant); and (3) invalid responses. The invalid responses were further categorized as: semantic error; syntactic error; other errors. Semantic errors represented final-word selections that did not fit the previous context in a semantically valid way, for example, if the final word had no semantic relationship with the subject or with the verb of the sentence (e.g., “A galinha põe milho”; "The chicken lays corn"). A syntactic error occurred when the final-word was syntactically invalid and violated grammatical rules, such as grammatical gender (male and female) and number (singular and plural) agreements (e.g., “A cabeleireira corta o^masculine article^ franja^feminine noun^”; “The hairdresser cuts the bangs”). Other-type errors included: (1) final-word with both semantic and syntactic violations (e.g., “O padre reza a^feminine article^ trabalho^masculine noun and incongruous^”–“The priest prays the work”); (2) using more than one final-word response (e.g., “O professor ensina o aluno a ler”; “The teacher teaches the student to read”) and (3) suffix error creating a word that does not exist (e.g., O carpinteiro faz uma carpintação”; “The carpenter makes a carpentertion”).

Two judges independently coded the responses. When there was a disagreement among them, they first tried to reach a consensus; when not possible, a third judge was involved and provided the final decision. All judges were experts in language assessment and were external to the research team. Inter-rater agreement was higher than 90%. Cloze probability level was established according to the criterion proposed by Block and Baldwin (2010) [18]: low cloze was defined as 0%–33% (.0-.33), medium cloze as 34%–66% (.34-.66), and high cloze as 67%–100% (.67–1).

### Data analyses

All statistical analyses were performed using the R statistical environment (RStudio, version 3.6.1, R Development Core Team, 2019) using the packages “glmmTMB” [44] and “effects” [45]. The statistical significance level was set at the conventional α = .05. The R script is available in Supporting Information S3 Rossi_R.R file.

The original dataset contains all participants’ responses to 100 context sentences. More specifically, each column corresponds to a different sentence and each row corresponds to a different participant (*N* = 361 rows). After identifying the most frequent word for each sentence, the following (100) dichotomous variables were added to the original dataset: occurrence (1 = “yes”, 0 = “no”) of the expected response in each cloze sentence; occurrence of idiosyncratic responses; and occurrence of errors, one for each error type (semantic, syntactic, and other errors).

All columns encoding information about the occurrence of expected words were used to construct a new simpler dataset (“S2 Dataset Rossi_sentences.csv”), which was used to assess the predictor effect of age on the generation of the most frequent word (S1 Table in S1 Appendix), for each sentence separately. The original dataset was also used to construct a coded long format dataset (“S2 Dataset Rossi.csv”), with *N*×100 rows. Moreover, in order to establish a comparison between error types, we constructed another dataset having *N*×100x3 rows and a single “Error” variable encoding information about the occurrence of invalid responses, whose error type was identified in another variable “Type” (semantic, syntactic, or other type).

To assess the predictor effect of age and of cloze probabilities on the generation of idiosyncratic and invalid responses, mathematical modeling tools were applied to each outcome, independently, using the “glmmTMB” R package [34]. Since all outcomes are dichotomous, mixed-effects logistic models with both participants and sentences random effects were performed. Indeed, logistic modeling is adequate to model a dichotomous outcome variable Y, i.e., a variable Y that only assumes two values: 0 or 1. Usually, the condition Y = 1 indicates an individual property in a population. In a logistic model, the odds of having Y = 1 are defined as the ratio [P(Y = 1)]/[P(Y = 0)] and reflect the likelihood for the condition Y = 1 (P means the probability; P(Y = 0) = 1-P(Y = 1)). Odds can be defined as the expected number of events divided by the expected number of non-events [46]. For example, consider a simple logistic model with a unique continuous predictor X, and assume that *b*_0_ and *b*_1_ are the model coefficients, *b*_1_ being associated with X. Then,

$$log\left(\left[P\left(Y=1\right)\right]/\left[P\left(Y=0\right)\right]\right)=b_{0}+b_{1}*\;X$$

This model allows to write the probability P(Y = 1) as:

$$exp\left(b_{0}+b_{1}*\;X\right)/\left[1+exp\left(b_{0}+b_{1}*\;X\right)\right]$$

In terms of interpretation, if *b*_1_ > 0 (*b*_1_ < 0), the coefficients of the model are interpreted as follows: one unit increase in X will increase (decrease) the odds of Y = 1 by exp(*b*_1_) times.

The two main datasets and the R script used to perform all analyses are available in the Supplementary Material (S1, S2 Data sets and S1 File).

## Results

### 1) Sentences analysis

As observed, 63 out of 100 sentence contexts met criteria for high cloze sentences, 30 were medium cloze probability, and 7 low cloze probabilities. Among the 63 high cloze sentences, 49 (77.8%) were from Pinheiro et al (2010) [31]. The cloze level was concordant in Brazil and Portugal databases for 31 out of 49 (83.7%) high-cloze sentences and for 7 low-cloze sentences. Among the remaining 26 medium cloze sentences there was no concordance between sentences from Brazil and Portugal (see Appendix–Table 1 in S1 Appendix).

For each sentence-context, we analyzed whether participants’ age influenced the generation of the most frequent word. To this purpose, logistic models were conducted, considering the dichotomous variable encoding information about the use of the most frequent word (1 = “yes”, 0 = “no”) as outcome. The results of these (one hundred) models are summarized in the Appendix (Table 1 in S1 Appendix), from high to low cloze probability. When inspecting age-related differences in cloze probability for each sentence, 63% of the sentences showed no age effects. Among these, 47 (74.6%) met criteria for high cloze probability, 10 (15.9%) had medium cloze probability, and 6 (9.5%) obtained low cloze probabilities.

We also examined whether cloze probabilities predicted the occurrence of idiosyncratic and invalid responses. Mixed-effects logistic models with both participants and sentences random effects were performed. At a first sight, this analysis might seem trivial since, for higher cloze probabilities, there should necessarily be less idiosyncratic and invalid responses. However, it is important to point out that a similar conclusion does not hold for lower cloze probabilities: when participants do not generate the most frequent word, they do not necessarily provide an idiosyncratic or invalid response, i.e. it may happen that another valid response has been given. Moreover, this analysis is also useful to compare differences between the effects of cloze probabilities on the three error types.

**Fig 1:**
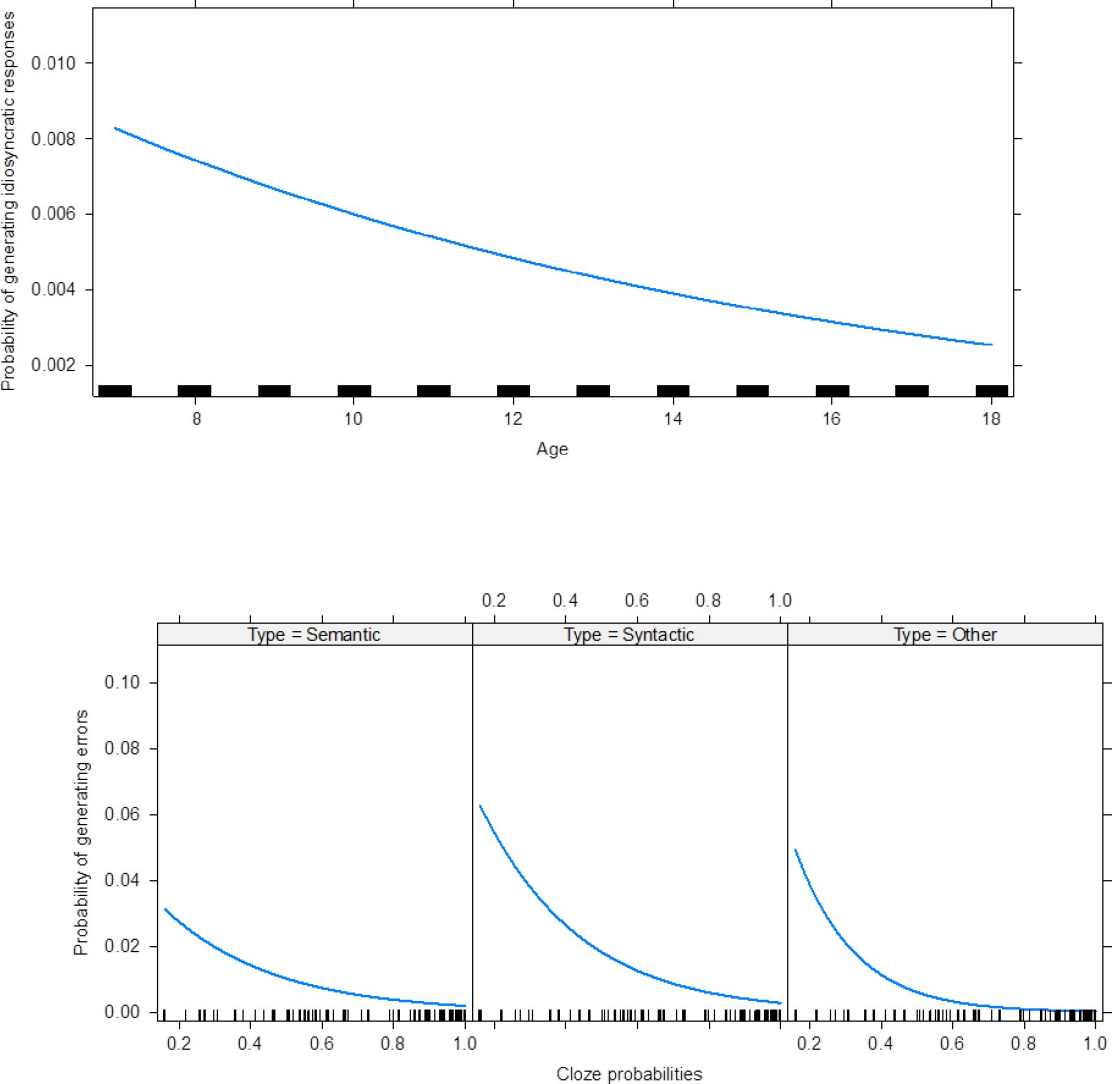
The predictor effect of cloze probabilities on the generation of idiosyncratic and invalid responses.

The results showed that higher cloze probabilities were associated with less idiosyncratic responses (estimate = -2.67, *p* < .001). In the case of invalid responses, and in order to compare the three error types, the interaction between cloze probabilities and the error type was selected as fixed effect. The results showed that higher cloze probabilities were associated with less semantic errors (estimate = -3.35), syntactic errors (estimate = -3.74), and other errors (estimate = -6.25), all cases with *p* < .001. In general, when compared to semantic errors, participants generated more syntactic errors (estimate = 0.80, *p* < .001) and other errors (estimate = 0.94, *p* < .001). However, as cloze probabilities increase, the decreased tendency of other error types is expected to be stronger (estimate = -2.93, *p* < .001). Appendix Table 2 in S1 Appendix summarizes the results of these two models and Fig 1 outlines these effects.

### 2) Participants’ performance analysis

After analyzing the cloze sentence features, we investigated the predictor effect of age on participants’ performance. More specifically, we examined participants’ ability to generate expected words, idiosyncratic responses, and errors (semantic, syntactic, and other type).

**Fig 2:**
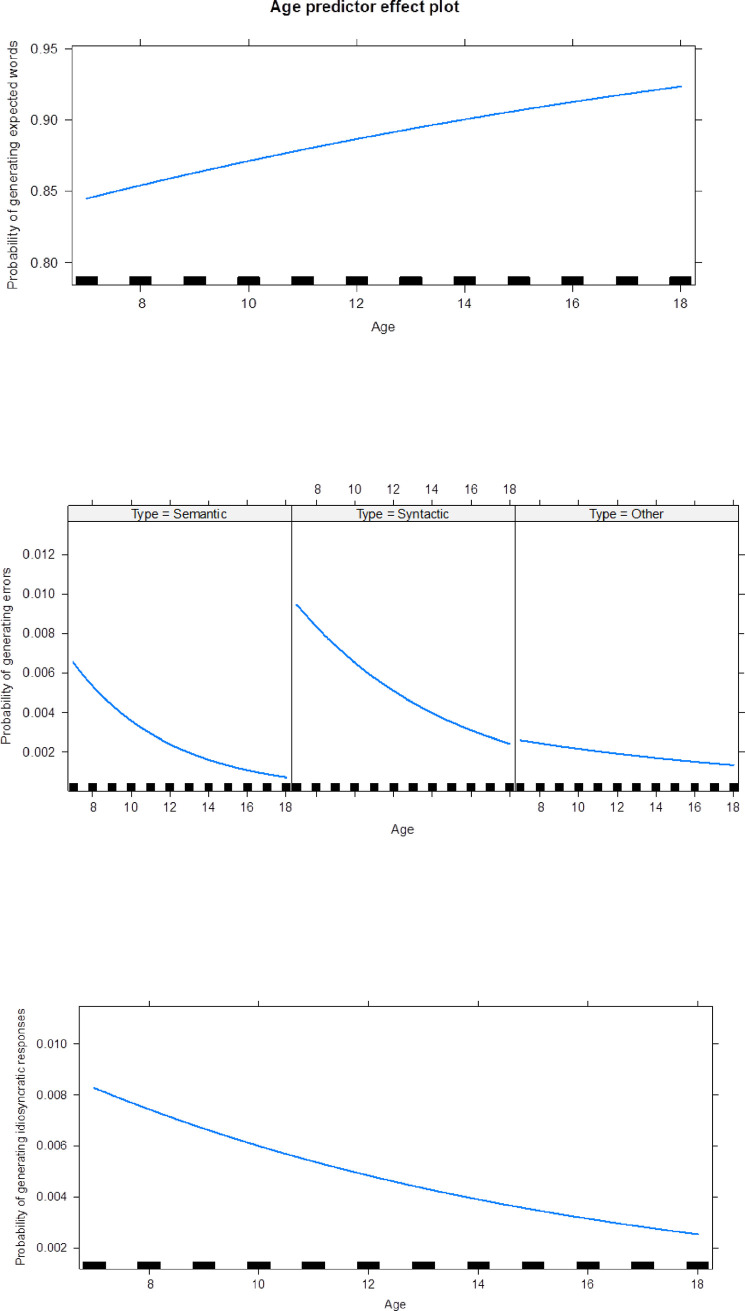
The predictor effect of age on the generation of expected answers, idiosyncratic responses, and errors.

Mixed-effects logistic models with both participants and sentences random effects were performed. For the most frequent word, the result showed a significant effect of age, with one year difference being associated with a 7% (exp(0.07) = 1.07, *p* < .001) increase in the odds of using the most frequent word. Fig 2 outlines this predictor effect and Appendix Table 3 in S1 Appendix summarizes the model.

We also examined whether age had a predictor effect on the generation of idiosyncratic and invalid responses. We observed that older participants are less likely to generate idiosyncratic responses (estimate = -0.11, *p* < .001); more precisely, if we compare two participants with one year difference, the odds of generating idiosyncratic responses is expected to be 10% smaller for the older [exp(-0.11) = 0.90 = 1–0.10, *p* = .005].

In the case of invalid responses, there were no significant differences between semantic and syntactic errors for the younger participants. The results showed that older participants are less likely to make semantic (estimate = -0.20) and syntactic errors (estimate = -0.12, *p* < .001), both with significance *p* < .001. For example, given two participants with a one year age difference, the odds of making semantic errors is expected to be 18% smaller for the older [exp(-0.20) = 0.82 = 1–0.18, *p* < .001]. However, the variation of these two types of errors over time differs, with older participants showing a stronger decrease of semantic errors (estimate = 0.08, *p* = 0.038). Table 2 in S1 Appendix summarizes the results of the three models and Fig 1 outlines these effects.

Participants made significantly less “other errors”, compared to semantic errors; for example, at 7 years old, the odds of making other error types is expected to be 82% smaller [exp(-1.74) = 0.18 = 1–0.82, *p* = .003], compared to the occurrence of semantic errors. The decreasing tendency of making errors over age was not significant for “other errors”, i.e., one additional year of age is expected to reduce the generation of “other errors” in a non-significant amount (estimate = -0.06, *p* = .098). However, this non-significant difference becomes significant when dichotomizing the age scale by considering two groups—children and adolescents. A significant difference on “other errors” was observed between the two groups; more specifically, the odds of making other errors is expected to decrease 37% for an adolescents [exp(-0.47) = 0.63 = 1–0.37, *p* = .028]. Notice that in this case we are not analyzing the effect of a one year old (continuous variable), but we are focusing on a group mean difference (categorical variable), which is a weaker constraint. The model describing these between group differences are shown in Appendix Table 4 in S1 Appendix (in order to better identify this difference in the results of the model, we used the reference level “other errors” of the predictor, so that the results are displayed using this error type as reference).

## Discussion

In the current study we have analysed a set of 100 sentence contexts and their cloze probabilities in order to develop a database for Brazilian Portuguese children and adolescents. Additionally, we have specified age-related effects and the predictor effects of age and cloze probabilities on the generation of idiosyncratic responses and errors (semantic, syntactic, and other errors). We observed that a high proportion of sentences (63%) met high-cloze probability criteria, in accordance with Pinheiro et al. (2010) [31]. As most sentence contexts were high-constraint contexts, the number of final word possibilities was limited, thereby eliciting the same most frequent final word [16, 17, 28, 47, 48]. Age was found to affect the probability to generate the most frequent word in 63% of the sentence contexts, whereas no age effects were observed for 47 (76%) contexts that met criteria for high cloze probability. These results are line with previous studies documenting the effects of developmental variables in a cloze task [1, 17, 32].

Of note, the observed age-related changes on cloze probability were not explained by the proportion of semantic or syntactic errors that were mainly observed in younger participants. For example, in sentence number 8 ("The fireman extinguishes the. . ."), adolescents often chose the word "incêndio" (*fire*) instead of “fogo” (*fire*). In English language, these words have the same orthographic representation (“fire"), hence context is necessary to define whether there is a fire situation outside control (represented by sentence number 8, for which the word “incêndio” would be more appropriate) or whether there is a minor event involving fire (for which the fireman do not need to be called, and the word “fogo” would be a more appropriate selection). In both cases, “fogo” instead of “incêndio” (fire in English) represent evidence of more general representations that were fitted within a specific context. These results are likely to represent developmental effects that reflect the refinement of the semantic system over age as a function of maturational processes and sociocultural factors (e.g., quality of language interactions, years of education) [31]. Accordingly, older participants are more likely to present maturational and sociocultural advantages over younger participants due to a longer and more diversified experience with the language system [31]. In fact, it is well established that an increase in the number of lexical items in semantic memory leads to richer semantic representations and vocabulary knowledge [49]. In the same line, vocabulary knowledge is important for the comprehension of single words, sentences, and narratives. Vocabulary size and vocabulary complexity are significantly increased with age [50–53]. Thus, the older participants are expected to use more refined words to fit the semantic context of a sentence [34, 54, 55], whereas children are expected to choose words reflecting more general representations, as suggested by our findings.

The current results are also consistent with those reported for European Portuguese regarding the number of idiosyncratic responses, semantic and syntactic errors [31]. In accordance, we observed that older participants were less likely to generate idiosyncratic responses and cloze errors, with no difference between semantic and syntactic errors. These findings are consistent with previous studies [31], where an increase in the commonality of responses with age and with years of education was documented [56]. Specifically, an increase of 5% per year of age was observed in the use of the most frequent final word for a given sentence context, and for each year a reduction between 10% and 16% is expected in the number of semantic errors, syntactic errors, and idiosyncratic responses.

Syntactic and semantic errors have been associated with limited semantic knowledge, with lexical gaps or weaker word representations [57]. We observed that most errors committed by younger participants were due to syntactic violations, caused by a violation of grammatical gender (e.g., in sentence number 92: “As pessoas comem no…", “People eat on the…”). In Portuguese grammar, the article before the noun will determine both gender and number; i.e., for a given sentence, a masculine and singular noun is required for the final word in the example provided (e.g., “prato”; “plate”). However, younger participants have selected the word “table” (“People eat at the table"), which represents a syntactic violation. In the case of English grammar, both final word “plate” (prato) and “table” (mesa) are possible. In some cases, younger participants committed a double semantic and syntactic violation. For example, in sentence number 16 (“O jardineiro rega o…”, The gardner waters the…”), younger participants sometimes completed the sentence with the word “flores” (flowers), which is a female and plural noun, even though the sentence requires a masculine and singular noun as final word.

Sociolinguistic studies conducted in Brazil have documented that plural matching is highly variable depending on the country [58]. The regular pattern of the verbal and nominal agreement is acquired over the years and is used when the individual spends more time in a formal educational context [58, 59]. Further, an electrophysiology study conducted in the Spanish language–which also has gender agreement between article and noun–showed an interaction between semantic congruity and gender agreement in the N400 component. A larger and more frontal N400 was observed for double violations (semantic and syntactic) compared to semantic violations alone. According to the authors, readers are able to anticipate and attend to the gender of both articles and nouns. Gender agreement and semantic congruity interact early in word processing to influence semantic integration of the noun into its sentence context [60]. Moreover, the grammatical gender agreement rule represents one of the most difficult language aspects to acquire during language development [61], which may account for the current pattern of findings.

In addition to semantic and syntactic errors, children showed two additional types of errors: (1) completion responses that exceeded the use of a single word; and (2) changes in sentence structure, such as replacing the grammatical gender of the article preceding the object plus gender agreement change. These types of errors suggest that younger children have the conceptual representation of the sentence, but fail to choose a specific word related with it. This finding may be related to weaker inhibitory mechanisms operating together with the constraint level of a given sentence [62]. Low-constraint sentences are associated with a larger number of valid final word choices as more features are activated intra-lexically in semantic memory, which brings additional demands to the inhibitory system [62]. Inhibitory control is necessary to suppress the activation of automatic responses that are irrelevant for the task [63]. It is well established that inhibitory skills increase with age [64, 65] and that children show a less effective use of inhibitory mechanisms compared to adolescents, which develop rapidly during early school years [66, 67]. As such, children are expected to present more difficulties in inhibiting lexical items that are semantically related to the sentence-context in their semantic memory, mainly for low-constraint sentences. In accordance, when we included age and cloze probability as predictors in the error analysis, we identified an increased number of errors for younger participants in low cloze sentence contexts. However, the interaction between age and a sentence’s constraint level appears to operate differently in adults [33] since age was not found to predict word selection in both high or low constraining sentence contexts.

Of note, the current study may also provide some insights into cross-cultural effects, namely by allowing comparisons of cloze probabilities between Brazilian and European Portuguese [31] children and adolescents. Specifically, most of our high cloze sentence contexts (31 of 49) displayed identical cloze probabilities to in Pinheiro et al. (2010) [31]. The remaining high cloze sentences from our study were classified as low cloze in Pinheiro et al. (2010) [31]. It is worth noting that the relationship between language and culture is complex. It is well known that the behaviors, beliefs, and customs of a given culture guide social interactions and rules that affect the communicative style and the development of language skills [68]. The current study did not control important cultural (educational level and socioeconomical status) and linguistic variables (lexical, syntactic, and morphological) to allow a more in-depth analysis of this relationship. The differences observed may be related to local, regional or more general country differences, impacting upon syntactic organization of sentences [69], as well as semantic lexicon development, and should be more systematically explored in future studies. Future studies should consider not only the cultural and linguistic differences between the two cultural contexts, but also the phonetic and orthographic differences between European *vs*. Brazilian Portuguese [38].

Finally, the results of the current study have important implications for experimental and clinical settings. The pool of sentence contexts described in our database and their cloze probabilities could be useful for future experimental studies probing language processing. In this realm, the selection of carefully controlled language stimuli often represents substantial challenge to researchers, relying on the use of norms for a given culture and age [70]. This database can be further used in electrophysiological and neuroimaging studies, in both typical and atypical developing populations in different developmental stages. In particular, investigating low and high cloze probability sentences with neuroimaging methods may shed light on the neural correlates of lexical and semantic processing [71]. Previous studies have documented a cloze probability effect both on the N400 event-related potential [72] and on the BOLD signal, specifically in the superior frontal gyrus and the inferior frontal gyrus [73].

A main limitation of the current study is the unbalanced age sample, characterized by a higher number of adolescents compared to children. Future studies should include bigger samples composed by different age intervals.

## Conclusions

The current study provides the first sentence database with cloze probabilities for Brazilian Portuguese. Both age and contextual constraint were found to predict the sentence completion response: final word selection was more consistent in older participants and for high-constraint sentence contexts. Even though similarities were observed for most sentence contexts in Brazilian and European Portuguese [31], some differences were also noted. These findings reinforce the importance of culture-specific norms. These norms are expected to stimulate experimental research on language processing at the sentence level.

## Supporting information

S1 Dataset Rossi_sentences. (CSV)

S2 Dataset Rossi. (CSV)

S1 File Rossi_R. (R)

S1 Appendix (DOCX)
