# Supplementary material for: Sentence contexts and cloze probabilities for Brazilian Portuguese children and adolescents
Source: PLoS One. 2020 Jul 30;15(7):e0236388. doi: 10.1371/journal.pone.0236388 (PMC7392299; doi:10.1371/journal.pone.0236388)
Supplement: S1 Appendix — (DOCX) [file pone.0236388.s004.docx]

**Appendix Table 1 – Individual Cloze sentences analysis: Cloze probabilities in descending order and the predictor effect of age on the probability to generate the associated expected word (or most frequent answer).**

| **CL** | | **Sentence** | | | **Cloze prob.** | **Age effect**  **(logistic model)** | | | | **Other observed valid answers** |
| --- | --- | --- | --- | --- | --- | --- | --- | --- | --- | --- |
| Portugal | Brazil |  | Sentence Context | Final  Word |  | Est. | SE | *p*-value | Age predictor effect plot |  |
|  | HC | 80 ^B^ | A cabelereira corta o…  (*The hairdresser cuts…*) | cabelo (hair) | 1.00 | .00 | .01 | 1 | 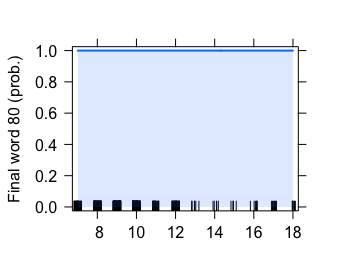 | --- |
| HC | HC | 3 ^P^ | O jogador chuta a…  (*The player kicks the*…) | bola  (ball) | .997 | -.84 | .61 | .1708 | 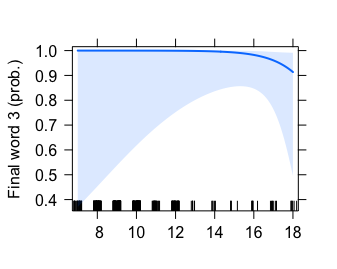 | --- |
| HC | HC | 35 ^P^ | A menina cala a…  (*The girl shuts the*…) | boca (mouth) | .997 | -.33 | .28 | .2437 | 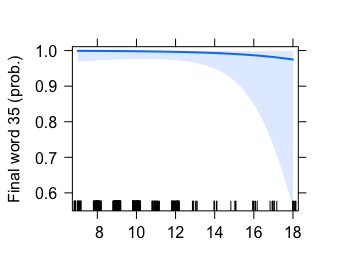 | colega (colleague) |
|  | HC | 98 ^B^ | O palhaço trabalha no…  (*The clown works in the*…) | circo (circus) | .997 | .26 | .64 | .687 | 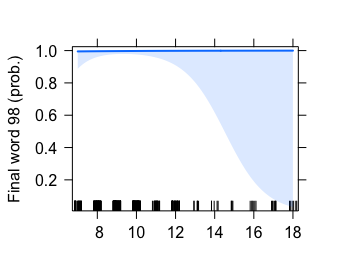 | picadeiro (stall) |
| HC | HC | 6 ^P-B^ | O pescador pesca um…  (*The fisherman fishes a/an*…) | peixe  (fish) | .989 | -.05 | .20 | .7866 | 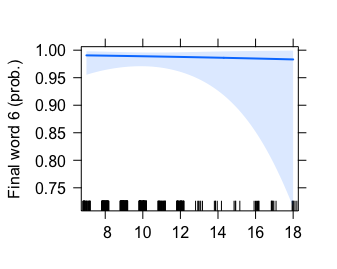 | polvo (octopus)  tubarão (shark) |
| HC | HC | 14 ^P^ | O livro conta uma…  (*The book tells a*…) | história  (story) | .989 | .38 | .37 | .299 | 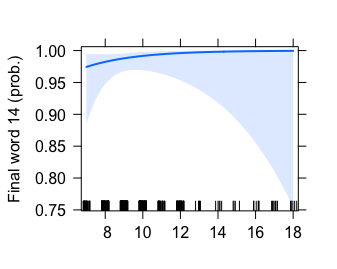 | piada (joke)  historinha (little story) |
| HC | HC | 17 ^P-B^ | A meia esquenta o…  (*The sock warms the...*) | pé  (foot) | .989 | .26 | .32 | .419 | 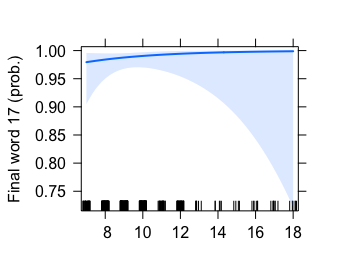 | --- |
| HC | HC | 21 ^P-B^ | A luva esquenta a…  (*The glove heats the*…) | mão  (hand) | .989 | .38 | .37 | .299 | 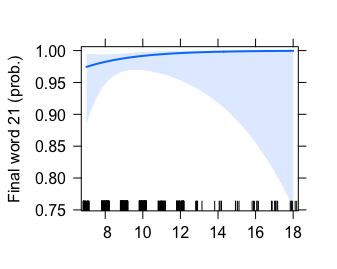 | --- |
| HC | HC | 30 ^P^ | O coelho come uma…  (*The rabbit eats a* …) | cenoura  (carrot) | .989 | .10 | .26 | .707 | 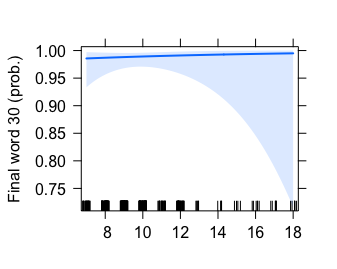 | ração (ration/food)  grama (grass) |
|  | HC | 77 ^B^ | A farmácia vende o…  (*The pharmacy sells the*…) | remédio (remedy) | .989 | -.09 | .19 | .6280 | 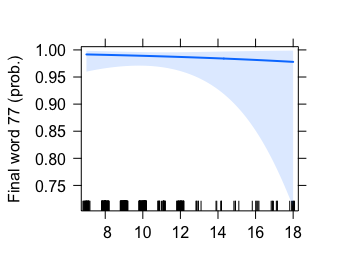 | medicamento (medicine)  produto (product) |
|  | HC | 81 ^B^ | O macaco come a…  (*The monkey eats the*…) | banana (banana) | .989 | .96 | .52 | .0637 | 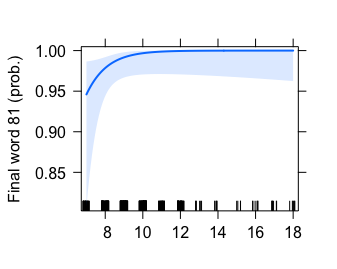 | fruta (fruit) |
|  | HC | 94 ^B^ | O sapateiro conserta o…  (*The shoemaker repairs the*…) | sapato (shoe) | .989 | .72 | .46 | .117 | 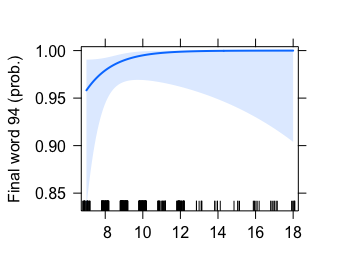 | par de sapatos (pair of shoes) |
| HC | HC | 1 ^P^ | A galinha põe um…  (*The chicken lays a/an*…) | ovo  *(egg)* | .986 | .60 | .39 | .117 | 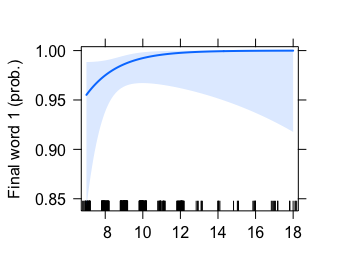 | pintinho  (chick) |
| HC | HC | 7 ^P^ | A menina penteia o…  *(The girl combs the…)* | cabelo  (hair) | .986 | .26 | .29 | .365 | 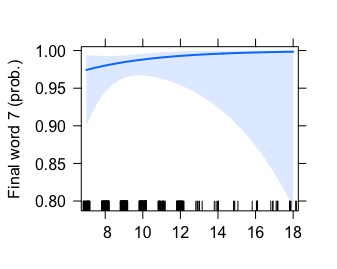 | menino (boy)  papagaio (parrot) |
| HC | HC | 40 ^P-B^ | A abelha produz o…  (*The bee produces*…) | mel  (honey) | .986 | .77 | .42 | .0689 | 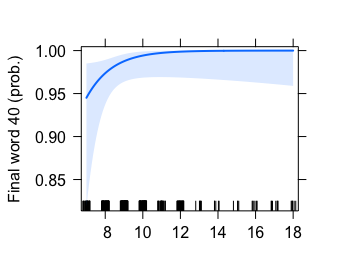 | nectar (nectar)  veneno (poison) |
|  | HC | 87 ^B^ | O motoqueiro acelera a…  (*The biker accelerates the*…) | moto (motorcycle) | .986 | .19 | .26 | .478 | 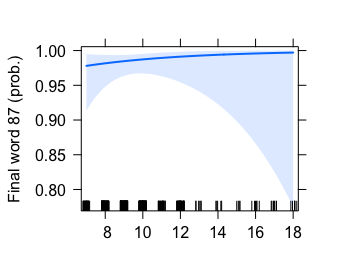 | --- |
| HC | HC | 11 ^P^ | A chave fecha a…  (*The key locks the*…) | porta  (door) | .983 | -.19 | .13 | .151 | 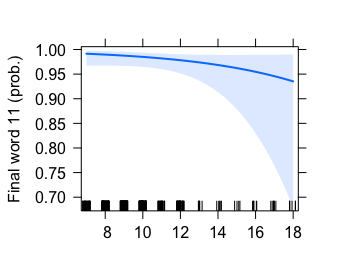 | casa (house)  fechadura (lock) |
| HC | HC | 8 ^P^ | O bombeiro apaga o…  (*The fireman extinguishes the*…) | fogo  (fire) | .981 | -.37 | .11 | .0010 *** | 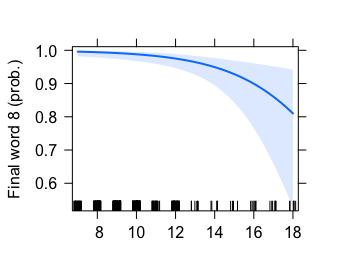 | incêndio  (conflagration) |
|  | HC | 85 ^B^ | A professora apaga a…  (*The teacher erases/turns off the*…) | lousa (blackboard) | .981 | .26 | .25 | .283 | 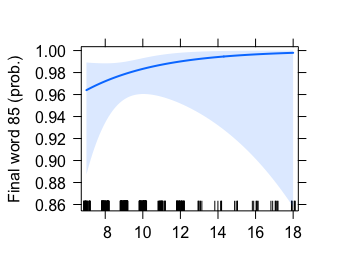 | luz (light) |
| HC | HC | 12 ^P^ | O padeiro faz o…  (*The baker makes the*…) | pão  (bread) | .978 | .63 | .31 | .0428 * | 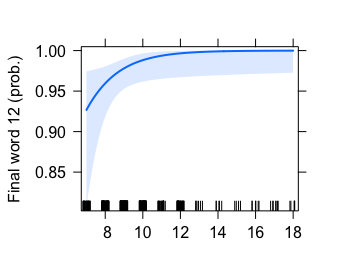 | bolo (cake)  trabalho (work) |
| LC | HC | 56 ^P-B^ | O pianista toca o...  (*The pianist plays the*…) | piano (piano) | .978 | .17 | .20 | .401 | 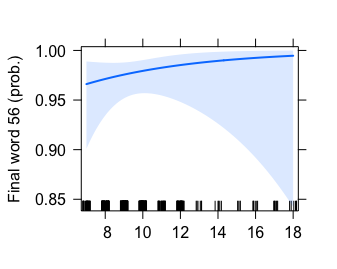 | instrumento (instrument) |
| HC | HC | 37 ^P^ | O cofre guarda o…  (*The safe box saves the*…) | dinheiro (money) | .975 | .03 | .15 | .8361 | 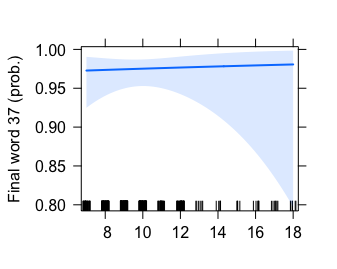 | diamante (diamond) |
| LC | HC | 27 ^P-B^ | O desenhista faz um…  (*The designer makes a*…) | desenho  (design) | .972 | .10 | .16 | .5488 | 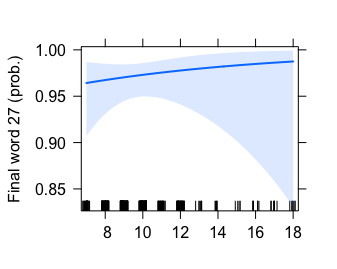 | quadro (picture)  painel (panel) |
|  | HC | 38 ^B^ | O sorveteiro vende o…  (*The ice cream man sells*…) | sorvete  (ice cream) | .972 | .61 | .28 | .0261 * | 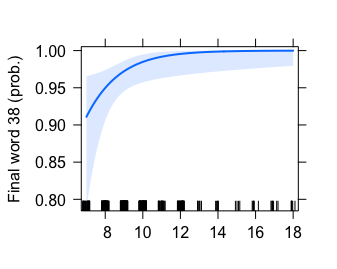 | picolé (popsicle) |
| LC | HC | 67 ^P-B^ | O carteiro entrega uma…  (*The postman delivers a/an*…) | carta (letter) | .972 | .10 | .16 | .5488 | 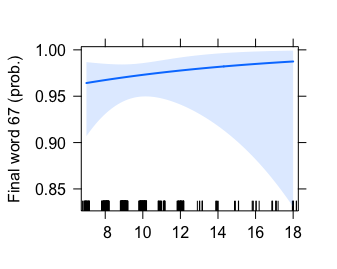 | correspondência (correspondnce)  encomenda  (order) |
| HC | HC | 20 ^P^ | O pedreiro constrói uma… (*Bricklayer builds a*…) | casa  (house) | .967 | -.07 | .11 | .5489 | 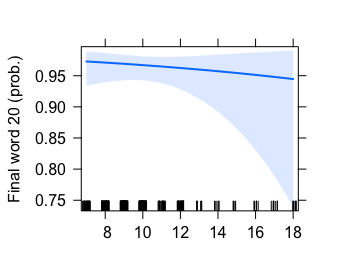 | parede (wall)  mansão (mansion) |
|  | HC | 89 ^B^ | O relógio marca a…  (*The clock shows the*…) | hora  (time) | .967 | .12 | .15 | .433 | 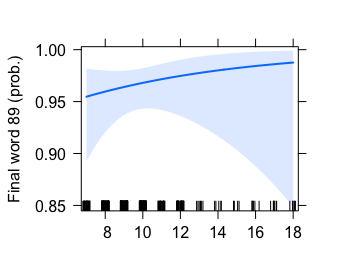 | --- |
|  | HC | 99 ^B^ | As pessoas dormem na…  (*People sleep in the*…) | cama (bed) | .964 | .24 | .17 | .177 | 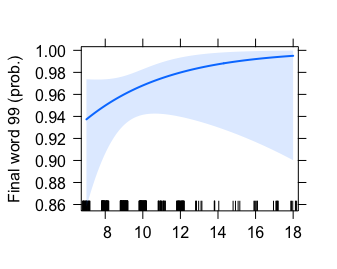 | casa (house)  sala (room) |
| HC | HC | 4 ^P-B^ | A torneira pinga muita…  (*The tap drips a lot of*…) | água  (water) | .958 | .07 | .13 | .568 | 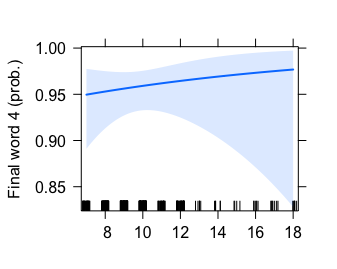 | gota  (drops) |
| HC | HC | 9 ^P^ | O dentista trata o…  *(The dentist treats the*…) | dente  (tooth) | .956 | .27 | .16 | .101 | 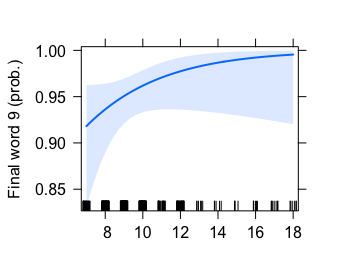 | homem (man)  paciente (patient) |
|  | HC | 82 ^B^ | O rato come o…  (*The mouse eats the*…) | queijo (cheese) | .956 | .15 | .14 | .276 | 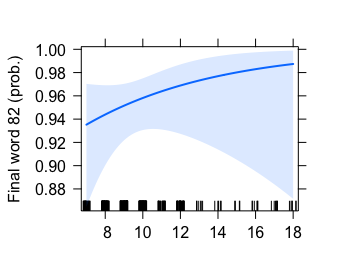 | veneno (poison)  inseto (insect) |
|  | HC | 76 ^B^ | O açougueiro corta a…  (*The butcher cuts the*…) | carne (meat) | .942 | .25 | .14 | .0736 | 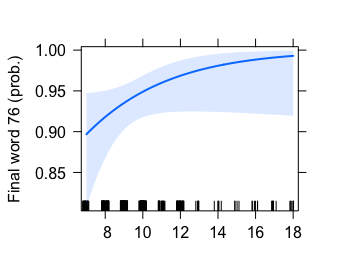 | linguiça (sausage)  galinha  (chicken) |
| HC | HC | 2 ^P-B^ | O aluno aponta o…  (*The student points the*...) | lapis  *(pencil)* | .936 | .03 | .10 | .7435 | 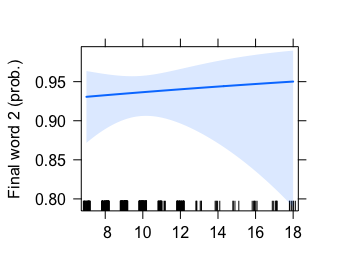 | dedo  (finger) |
| HC | HC | 23 ^PB^ | O mecânico conserta o…  (*The mechanical repairs the*…) | carro  (car) | .936 | -.04 | .09 | .6794 | 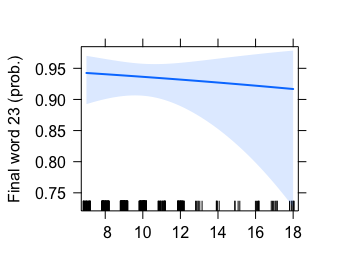 | automóvel (automobile)  veículo (vehicle) |
| LC | HC | 19 ^P^ | A girafa levanta o…  (*The giraffe raises the*…) | pescoço  (neck) | .934 | .18 | .13 | .143 | 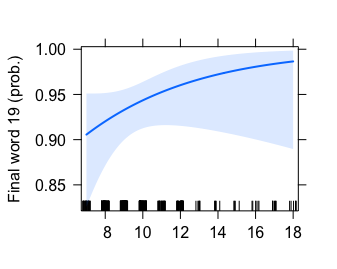 | rabo (tail)  pé (foot) |
| LC | HC | 57 ^P-B^ | O cachorro abana o…  (*The dog wags the*…) | rabo  (tail) | .931 | .28 | .13 | .0387 * | 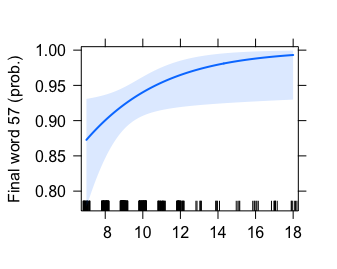 | rabinho (small tail)  mosquito (mosquito) |
|  | HC | 86 ^B^ | O bebê chupa a…  (*The baby sucks the*…) | chupeta (pacifier) | .931 | .13 | .11 | .235 | 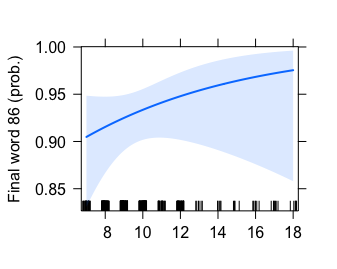 | água (water)  bala (candy) |
| HC | HC | 5 ^P^ | O varredor utiliza a…  (*The sweeper uses the*…) | vassoura  (broom) | .928 | .47 | .16 | .0032 ** | 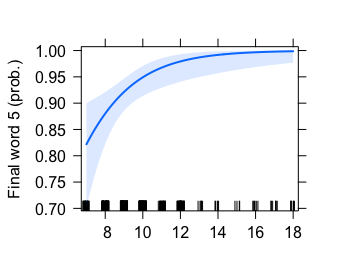 | água (water)  pá (shovel) |
|  | HC | 93 ^B^ | A minhoca vive na…  (*The worm lives in the*…) | terra  (soil) | .928 | .28 | .13 | .0348 * | 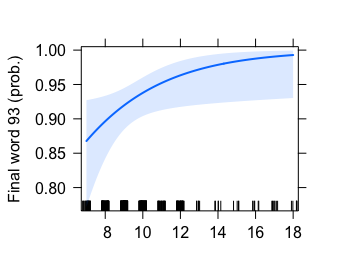 | areia (sand)  mata (wood) |
| HC | HC | 18 ^B^ | O menino nada na…  (*The boy swims in the*…) | piscina  (swimming pool) | .914 | .42 | .14 | .0028 ** | 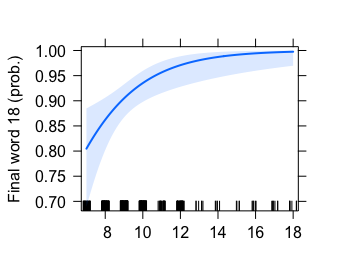 | água (water)  lagoa (pool/pond) |
| HC | HC | 13 ^P^ | O gato caça um…  (*The cat hunts a*…) | rato  (mouse) | .900 | .04 | .08 | .6130 | 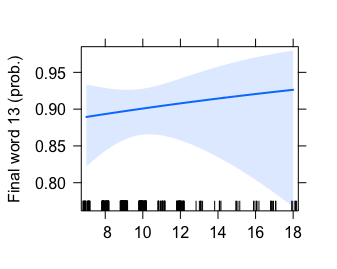 | passarinho (birdie)  peixe (fish) |
| LC | HC | 24^P^ | A mãe lê um…  (*The mother reads a*…) | livro  (book) | .900 | .16 | .10 | .0897 | 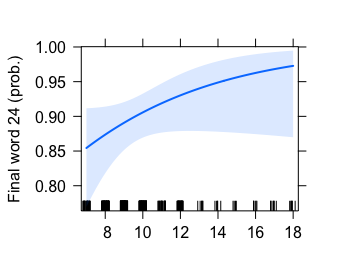 | conto (tale)  jornal (newspaper) |
| HC | HC | 28 ^P^ | O rádio faz muito…  (*The radio makes a lot of*…) | barulho  (noise) | .900 | .13 | .09 | .161 | 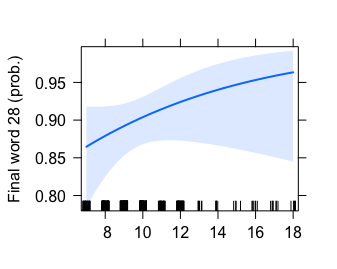 | som (sound)  sucesso (success) |
| LC | HC | 47 ^P^ | A comida enche a...  (*The food fills the*…) | barriga (belly) | .898 | .05 | .08 | .5691 | 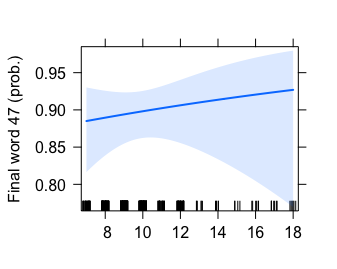 | boca (mouth)  panela (pan) |
| LC | HC | 88 ^P-B^ | A telefonista atende o…  (*The telephonist answers the*…) | telefone (phone) | .898 | .30 | .11 | .0087 ** | 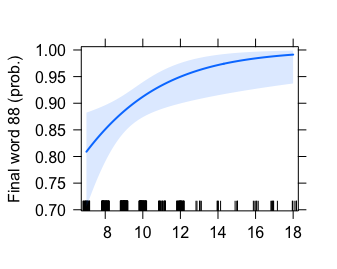 | cliente (costumer)  paciente (patient) |
| HC | HC | 34 ^P-B^ | O porteiro abre o…  (*The doorman opens the*…) | portão (gate) | .895 | .45 | .13 | .0006 *** | 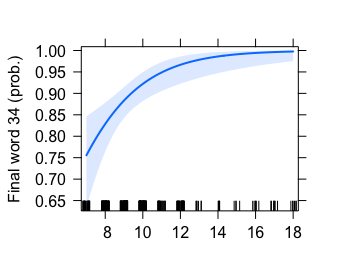 | elevador (elevator)  prédio (building) |
| LC | HC | 55 ^P^ | A bicicleta atravessa a…  (*The bike crosses the*…) | rua  (street) | .894 | -.01 | .07 | .8563 | 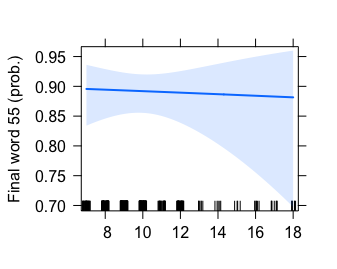 | pista (runway)  calçada (sidewalk) |
| HC | HC | 45 ^P^ | A galinha come o...  (*The chicken eats the*…) | milho (corn) | .889 | .29 | .11 | .0079 ** | 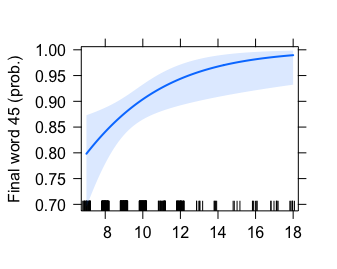 | trigo (wheat)  alpiste (birdseed) |
| LC | HC | 43 ^P^ | O balde transporta muita…  (*The bucket carries a lot of*…) | água (water) | .886 | .14 | .09 | .114 | 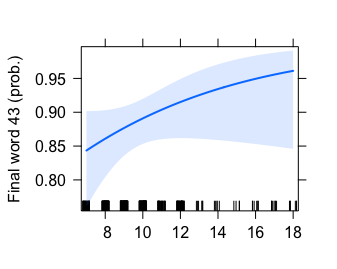 | roupa (clothing)  terra (soil) |
|  | HC | 90^B^ | A manicure faz a…  (*The manicure works/does the*…) | unha  (nail) | .873 | .12 | .08 | .137 | 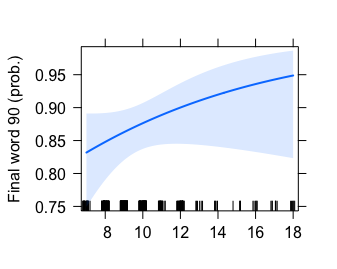 | mão (hand)  massagem (massage) |
| HC | HC | 49 ^P^ | A pulseira enfeita o…  (*The bracelet adorns the*…) | braço (arm) | .859 | .03 | .07 | .615 | 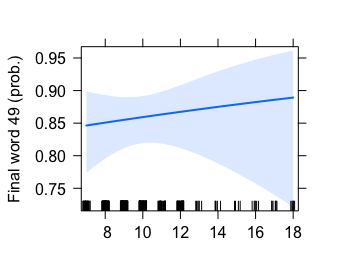 | pulso (wrist)  corpo (body) |
| LC | HC | 51 ^P^ | A empregada arruma a…  (*The maid tidies the*…) | casa  (home) | .859 | .18 | .08 | .0308 * | 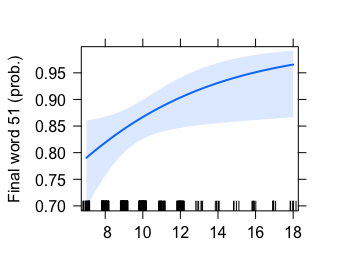 | cama (bed)  sala (room) |
| HC | HC | 25 ^P^ | A noiva compra um…  (*The bride buys a*…) | vestido  (dress) | .848 | .17 | .08 | .0323 * | 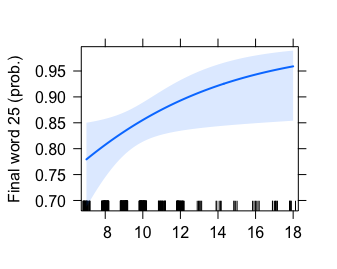 | bouquet (bouquet)  anel (ring) |
| HC | HC | 64 ^P-B^ | O motorista dirige o…  (*The chauffeur drives the*…) | carro  (car) | .848 | .07 | .07 | .301 | 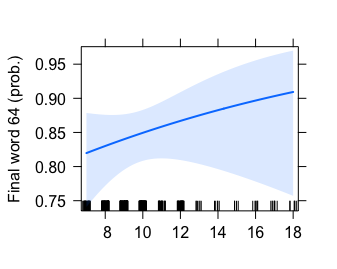 | caminhão (truck)  onibus (bus) |
| LC | HC | 16 ^P^ | O jardineiro rega o...  (*The gardener waters the*…) | jardim  (garden) | .814 | .20 | .08 | .0091 ** | 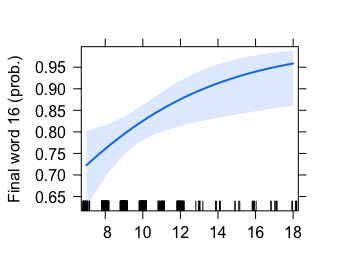 | quintal (backyard)  vaso (flowerpot) |
| HC | HC | 54 ^P-B^ | O padre reza a…  (*The priest prays the/to*…) | missa (mass) | .814 | .22 | .08 | .0055 ** | 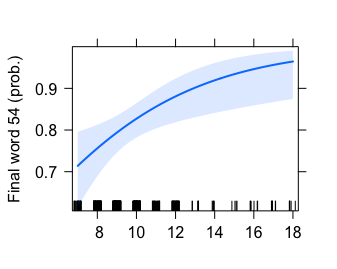 | Deus (God)  oração (prayer) |
| HC | HC | 10 ^P^ | A criança bebe o…  (*The child drinks*…) | leite  (milk) | .798 | .15 | .07 | .0289 * | 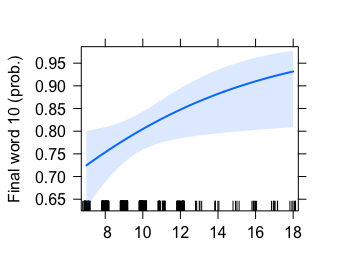 | suco (juice)  guaraná (guarana juice) |
| LC | HC | 39 ^P^ | O professor ensina o…  (*The teacher teaches the*…) | aluno (student) | .789 | .07 | .06 | .242 | 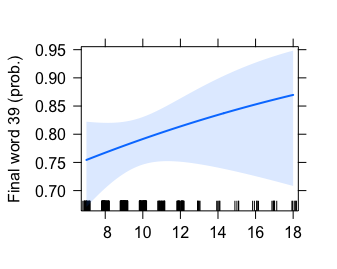 | português (Portuguese language)  trabalho  (work) |
|  | HC | 91 ^B^ | Os animais vivem na…  (*The animals live in the*…) | floresta (forest) | .731 | .07 | .05 | .207 | 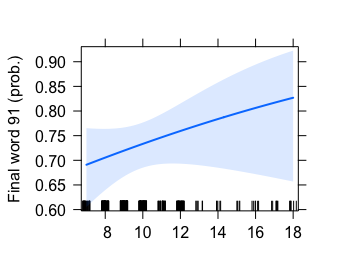 | selva (jungle)  mata (wood) |
| LC | HC | 70 ^P-B^ | A escritora escreve um…  (*The writer writes a* …) | livro  (book) | .729 | .05 | .05 | .341 | 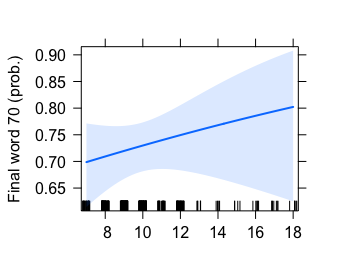 | texto (text)  poema (poem) |
| 55(HC) | HC | 32 ^P^ | O árbitro segura o…  (*The referee holds the*…) | apito  (whistle) | .709 | .44 | .08 | <.0001 *** | 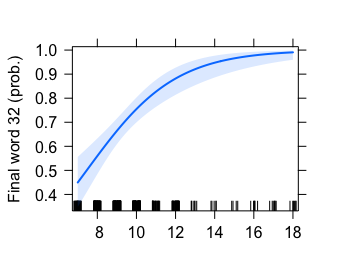 | cartão (card)  jogo (match) |
| LC | HC | 53 ^P^ | A tesoura recorta a…  (*The scissors cut the*…) | folha (sheet) | .709 | -.08 | .05 | .1169 | 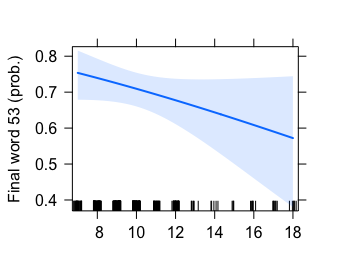 | roupa (clothing)  revista (magazine) |
| LC | HC | 36 ^P-B^ | O turista faz um…  (*The tourist takes/does a*…) | passeio (walk) | .673 | .14 | .06 | .0129 * | 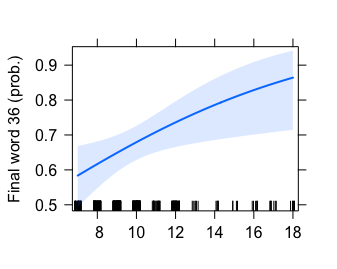 | turismo (tourism)  trajeto (journey) |
|  | MC | 78 ^B^ | O policial prende o…  (*The officer arrests the*…) | ladrão (thief) | .665 | -.03 | .05 | .5750 | 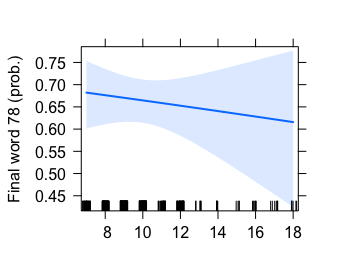 | bandido (bandit)  assaltante (robber) |
| LC | MC | 41 ^P^ | A senhora cheira um…  (*The woman smells a*…) | perfume (perfume) | .662 | .17 | .06 | .0028 ** | 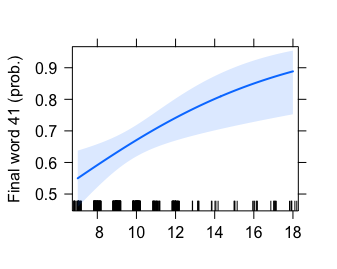 | bolo (cake)  cravo (carnation) |
| LC | MC | 63 ^P-B^ | A batedeira bate o…  (*The mixer beats the*…) | bolo  (cake) | .662 | .13 | .05 | .0159 * | 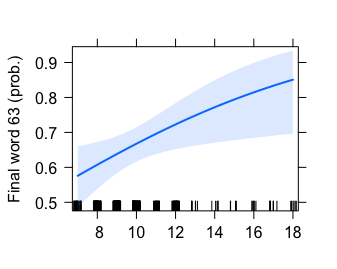 | ovo (egg)  suco (juice) |
| LC | MC | 58 ^P^ | O avestruz esconde a…  (*The ostrich hides its*…) | cabeça (head) | .659 | .19 | .06 | .0012 ** | 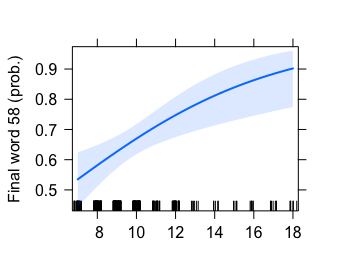 | pena (feather)  asa (wing) |
|  | MC | 75 ^B^ | A cozinheira tempera a…  (*The cook tempers the*…) | comida (food) | .632 | .11 | .05 | .0349 * | 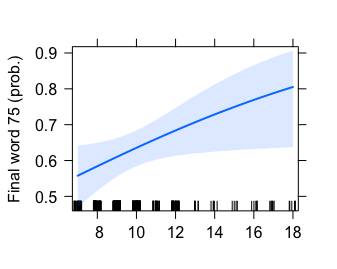 | salada (salad)  carne (meat) |
|  | MC | 96 ^B^ | O papai lê o…  (*Dad reads the*…) | livro  (book) | .618 | -.08 | .05 | .0809 | 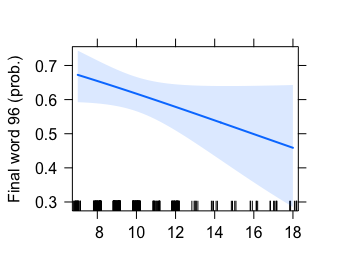 | jornal (newspaper)  gibi  (comic book) |
|  | MC | 100 ^B^ | O cinto segura a…  (*The belt holds the*…) | calça (pants) | .615 | .07 | .05 | .126 | 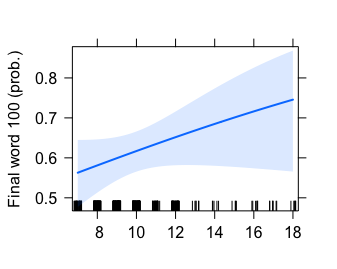 | pessoa (person)  criança (child) |
| HC | MC | 22 ^P-B^ | O menino calça o…  (*The boy shoes the*…) | sapato  (shoe) | .612 | -.17 | .05 | .0004 *** | 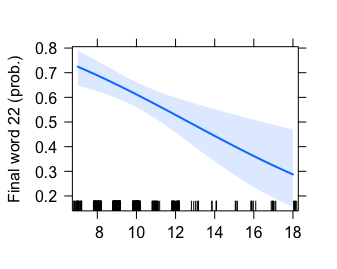 | tenis (tennis shoe)  chinelo (slipper) |
| LC | MC | 68 ^P^ | O coelho ultrapassa a…  (*The rabbit exceeds the*…) | tartaruga (turtle) | .612 | .10 | .05 | .0386 * | 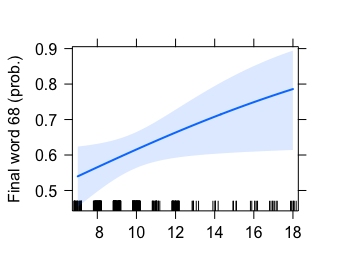 | raposa (fox)  lebre (hare) |
|  | MC | 79 ^B^ | A enfermeira cuida do…  (*The nurse takes care of the*…) | paciente (patient) | .593 | .12 | .05 | .0192 * | 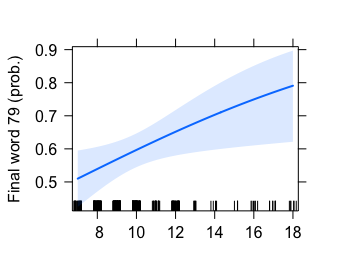 | doente (sick)  homem (man) |
|  | MC | 97 ^B^ | O ladrão está na…  (*The thief is in the*…) | cadeia  (jail) | .593 | .03 | .05 | .501 | 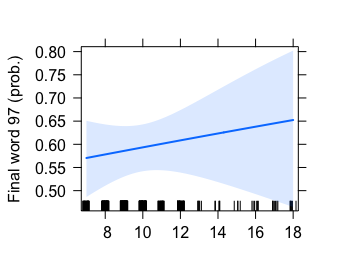 | prisão (prison)  casa (house) |
|  | MC | 84 ^B^ | O peixe nada no…  (*The fish swims in the*…) | rio  (river) | .582 | .01 | .05 | .770 | 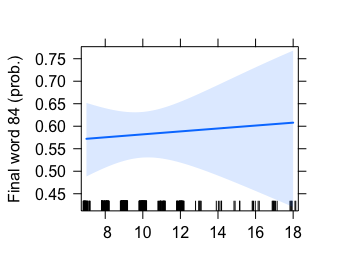 | mar (sea)  aquário (aquarium) |
| LC | MC | 42 ^P^ | A mão espreme uma…  (*The hand squeezes a/an*…) | laranja (orange) | .573 | .05 | .05 | .290 | 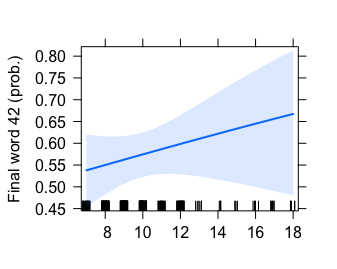 | espinha (pimple)  esponja (sponge) |
| HC | MC | 44 ^P^ | O papagaio diz uma…  (*The parrot says a*…) | palavra (word) | .573 | .08 | .05 | .110 | 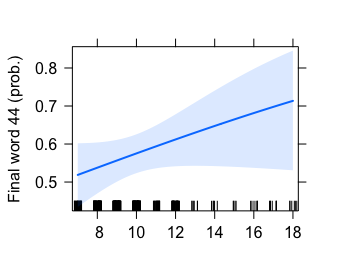 | frase (statement)  piada (joke) |
| HC | MC | 29 ^P^ | A cozinheira acende o...  (*The cook lights the*…) | fogão  (stove) | .562 | .17 | .05 | .0011 ** | 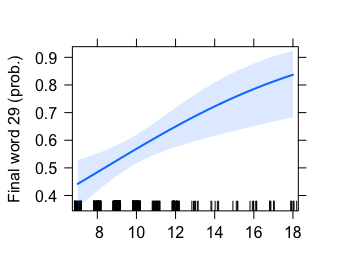 | fogo (fire)  forninho (little stove) |
| HC | MC | 15 ^P^ | A almofada enfeita a…  (*The cushion graces the*…) | sala  (room) | .560 | -.04 | .05 | .409 | 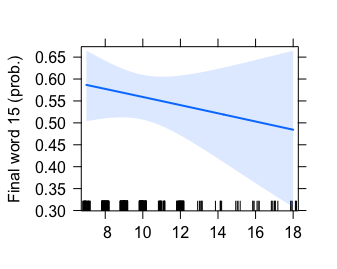 | cama (bed)  casa (house) |
| LC | MC | 26 ^P^ | O pintor pinta um…  (*The painter paints a*…) | quadro  (picture) | .551 | .12 | .05 | .0162 * | 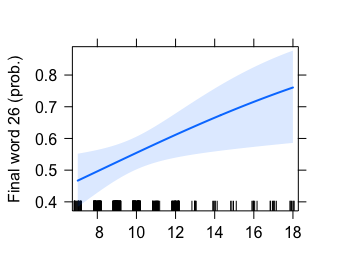 | muro (wall)  carro (car) |
| LC | MC | 62 ^P^ | O vento arrasta a…  (*The wind drags the*…) | folha  (leaf) | .537 | -.09 | .05 | .0500 * | 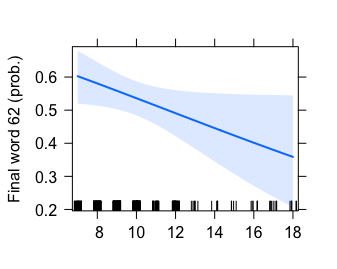 | sujeira (dirtiness)  poeira  (dust) |
| HC | MC | 59 ^P-B^ | O lixeiro varre a…  (*The garbage collector sweeps the*…) | rua  (street) | .535 | .11 | .05 | .0218 * | 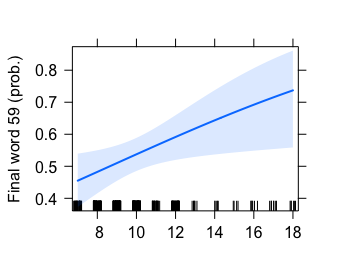 | calçada (sidewalk)  sujeira (dirtiness) |
|  | MC | 92 ^B^ | As pessoas comem no…  (*People eat on/in/at (the)*…) | prato (plate) | .518 | -.12 | .05 | .0098 ** | 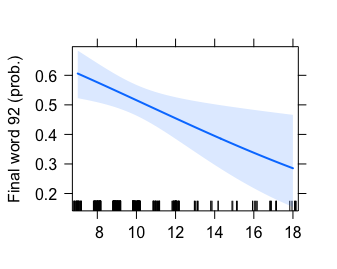 | restaurante (restaurant)  almoço (lunch) |
| LC | MC | 33 ^P^ | A esponja absorve a…  (*The sponge absorbs the*…) | água (water) | .507 | .20 | .05 | .0001 *** | 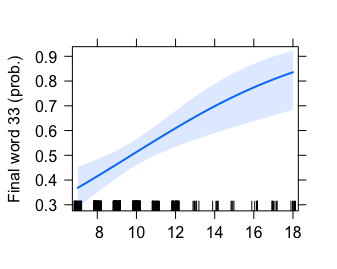 | espuma (foam)  sujeira (dirtiness) |
| LC | MC | 46 ^P^ | A mulher veste uma…  (*The woman wears (a)*…) | roupa (clothing) | .501 | -.10 | .05 | .0338 * | 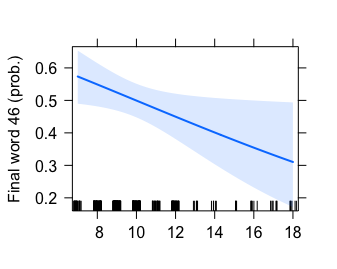 | saia (skirt)  blusa (shirt) |
|  | MC | 74 ^B^ | O jornalista transmite a…  (*The journalist transmits the*…) | notícia (news) | .465 | .15 | .05 | .0024 ** | 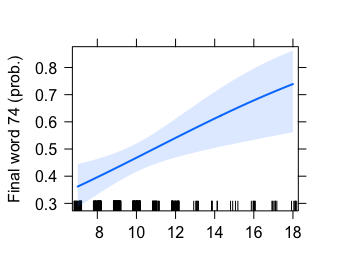 | reportagem (news report)  informação  (information) |
| LC | MC | 61 ^P^ | A raposa persegue a...  (*The fox chases the*…) | galinha (chicken) | .463 | .11 | .05 | .0234 * | 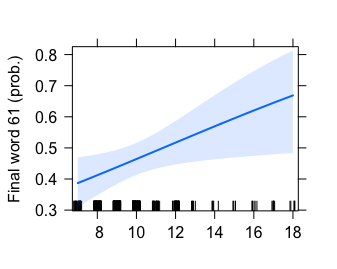 | lebre (hare)  caça (hunt) |
|  | MC | 95 ^B^ | O menino escuta a…  (*The boy listens to the/his*…) | música (music) | .463 | .25 | .05 | <.0001 *** | 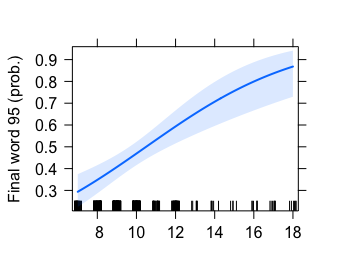 | mãe (mother)  professora (teacher) |
| LC | MC | 52 ^P^ | A mãe folheia a...  (*The mother leafs the*…) | revista (magazine) | .438 | .06 | .05 | .1724 | 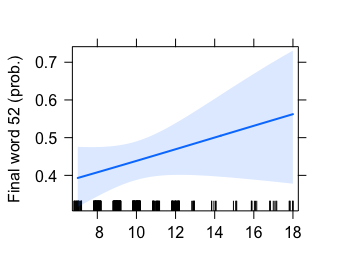 | página (page)  lista (list) |
| LC | MC | 31 ^P^ | A secretária atende o…  (*The secretary answers the*…) | telefone  (phone) | .413 | .17 | .05 | .0004 *** | 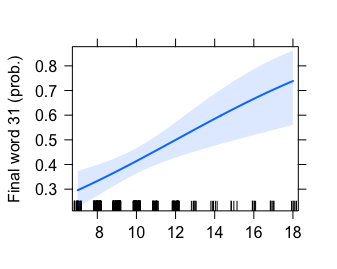 | cliente (customer)  paciente (patient) |
|  | MC | 83 ^B^ | O carro para no…  (*The car stops in the*…) | estacionamento (parking) | .380 | .04 | .05 | .3835 | 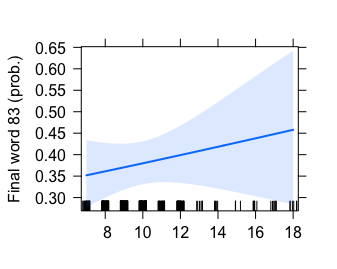 | sinal (traffic signal)  trânsito (traffic) |
| LC | MC | 65 ^P^ | O bar vende muito…  (*The bar sells a lot of*…) | doce (sweets) | .357 | .04 | .05 | .4445 | 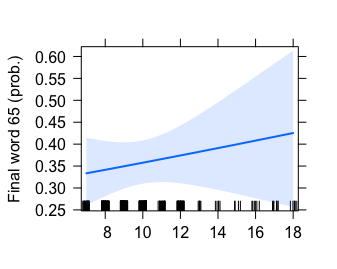 | salgado (salty food)  refrigerante (soft drinks) |
| LC | MC | 72 ^P^ | O agricultor planta uma…  (*The farmer plants a*…) | flor  (flower) | .355 | -.22 | .06 | .0002 *** | 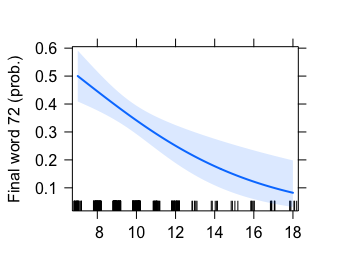 | planta (plant)  árvore (tree) |
| LC | LC | 60 ^P^ | O aluno levanta o...  (*The student raises his*…) | braço  (arm) | .307 | .09 | .05 | .0644 | 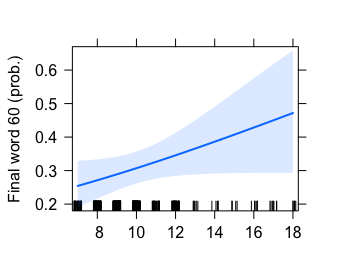 | dedo (finger)  caderno (notebook) |
| LC | LC | 73 ^P^ | O menino esvazia o…  (*The boy empties the*…) | balde (bucket) | .296 | -.03 | .05 | .580 | 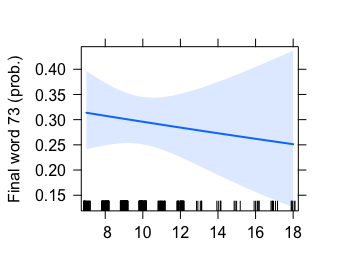 | copo (glass)  cofre (safe box) |
| LC | LC | 50 ^P^ | A costureira usa o…  (*The seamstress uses the*…) | tecido (tissue) | .271 | .06 | .05 | .2011 | 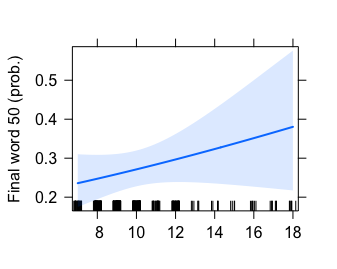 | pano (cloth)  dedal (thimble) |
| HC | LC | 66 ^P^ | O espelho mostra a…  (*The mirror shows the*…) | cara  (face) | .258 | -.11 | .06 | .0647 | 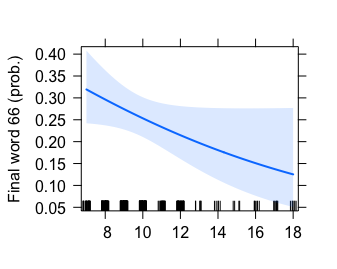 | pessoa (person)  imagem (image) |
| LC | LC | 71 ^P^ | O homem pendura o…  (*The man hangs the*…) | casaco (coat) | .219 | .04 | .05 | .443 | 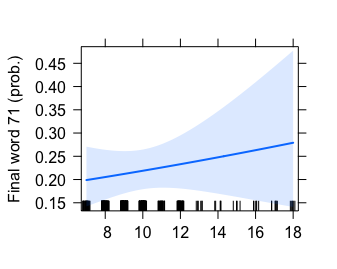 | chapéu (hat)  quadro (picture) |
| LC | LC | 69 ^P^ | O carpinteiro faz uma…  (*The carpenter makes a*…) | mesa (table) | .161 | .16 | .05 | .0042 ** | 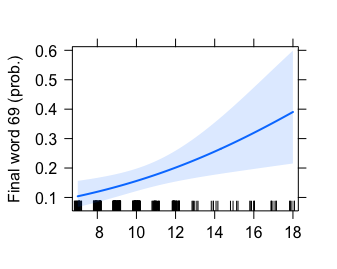 | casa (house)  cadeira (chair) |
| LC | LC | 48 ^P^ | A cama tem uma...  (*The bed has a*...) | cabeceira  (headbord) | .158 | .08 | .06 | .182 | 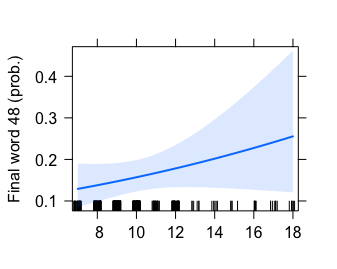 | almofada (pillow)  colcha (quilt) |

*Legend:* CL = Constraint level; P = sentences from Portugal; B = sentences from Brazil; P-B = sentences from Portugal with semantic adaptation to Brazilian vocabulary; HC = High cloze; MC = Medium cloze; LC = Low Cloze. Shaded area is a pointwise 95% confidence band for the fitted values, based on standard errors and computed from the covariance matrix of the fitted regression coefficients.

**Appendix Table 2 - The predictor effect of cloze probabilities on the generation of idiosyncratic and invalid answers.**

| Table 2. The predictor effect of cloze probabilities on the generation of idiosyncratic and invalid answers. | | | | |
| --- | --- | --- | --- | --- |
| *Model* |  |  |  |  |
| Variable | Estimate | SE | z-value | Pr(>\|z\|) |
| *Model 1: Idiosyncratic responses* | | | | |
| Intercept | -3.07 | .29 | -10.49 | <.0001 *** |
| Cloze prob. | -2.67 | .39 | -6.91 | <.0001 *** |
| *Model 2: Invalid answers* | | | | |
| Intercept | -2.90 | .39 | -7.43 | <.0001 *** |
| Syntactic | 0.80 | .17 | 4.60 | <.0001 *** |
| Other errors | 0.94 | .21 | 4.42 | <.0001 *** |
| Cloze prob. | -3.35 | .52 | -6.46 | <.0001 *** |
| Cloze prob. x Syntactic | -0.42 | .27 | -1.55 | 0.12 |
| Cloze prob. x Other errors | -2.93 | .39 | -7.49 | <.0001 *** |
|  |  |  |  |  |
| *Note*. SE – Standard error. Significance: ****p* < .001. | | | | |

**Appendix Table 3 - The predictor effect of age on the generation of expected words, idiosyncratic responses, and errors.**

| Table 3. The predictor effect of age on the generation of expected words, idiosyncratic responses, and errors. | | | | |
| --- | --- | --- | --- | --- |
| *Model* |  |  |  |  |
| Variable | Estimate | SE | z-value | Pr(>\|z\|) |
| *Model 3: Expected words* | | | | |
| Intercept | 1.19 | .22 | 5.46 | <.0001 *** |
| Age | 0.07 | .01 | 7.01 | <.0001 *** |
| *Model 4: Idiosyncratic responses* | | | | |
| Intercept | -4.03 | .32 | -12.46 | <.0001 *** |
| Age | -0.11 | .03 | -3.51 | 0.0005 *** |
| *Model 5: Invalid answers* | | | | |
| Intercept | -3.62 | .37 | -9.86 | <.0001 *** |
| Syntactic | -0.16 | .35 | -0.47 | 0.6390 |
| Other errors | -1.91 | .43 | -4.39 | <.0001 *** |
| Age | -0.20 | .03 | -5.75 | <.0001 *** |
| Age x Syntactic | 0.08 | .04 | 2.07 | 0.0383 * |
| Age x Other errors | 0.14 | .04 | 3.13 | 0.0018 ** |
|  |  |  |  |  |
| *Note*. SE – Standard error. Significance: **p* < .05, ***p* < .01, ****p* < .001. | | | | |

**Appendix Table 4 – Differences between children and adolescents on the generation of errors.**

| Table 4. Differences between children and adolescents on the generation of errors. | | | | |
| --- | --- | --- | --- | --- |
| *Model 6: Invalid answers* |  |  |  |  |
| Variable | Estimate | SE | z-value | Pr(>\|z\|) |
|  | | | | |
| Intercept | -6.06 | .18 | -34.44 | <.0001 *** |
| Semantic | 0.56 | .09 | 6.22 | <.0001 *** |
| Syntactic | 1.13 | .08 | 13.53 | <.0001 *** |
| Adolescents Group | -0.47 | .21 | -2.20 | 0.0277 * |
| Adolescents Group x Semantic | 0.12 | .24 | 0.53 | 0.5992 |
| Adolescents Group x Syntactic | 0.02 | .22 | 0.08 | 0.9387 |
|  |  |  |  |  |
| *Note*. SE – Standard error. Significance: **p* < .05, ****p* < .001. | | | | |
